# Colonization of larval zebrafish (*Danio rerio*) with adherent-invasive *Escherichia coli* prevents recovery of the intestinal mucosa from drug-induced enterocolitis

**DOI:** 10.1128/msphere.00512-23

**Published:** 2023-11-16

**Authors:** Erika Flores, Soumita Dutta, Rachel Bosserman, Ambro van Hoof, Anne-Marie Krachler

**Affiliations:** 1 Microbiology and Infectious Diseases Program, MD Anderson Cancer Center UTHealth Graduate School of Biomedical Sciences, University of Texas, Houston, Texas, USA; 2 Department of Microbiology and Molecular Genetics, The University of Texas Health Science Center at Houston, Houston, Texas, USA; University of Kentucky College of Medicine, Lexington, Kentucky, USA

**Keywords:** zebrafish, colitis, AIEC, intestinal colonization

## Abstract

Inflammatory bowel disease (IBD) is a broad term for a range of chronic intestinal disorders, including Crohn’s disease and ulcerative colitis. The global prevalence of IBD is rising, with over one million patients affected in the United States alone. Adherent-invasive *Escherichia coli* (AIEC) is a pathobiont frequently found in IBD biopsies. AIEC adhere to and invade epithelial cells, and can survive inside phagocytes *in vitro*. However, how AIEC contribute to IBD *in vivo* remains unclear. Here, we established a larval zebrafish (*Danio rerio*) model to study the interplay between pre-existing intestinal inflammation and AIEC colonization of the gut. We used the pro-inflammatory drug dextran sulfate sodium (DSS) to induce intestinal inflammation. This was followed by food-borne infection of larvae with AIEC using the protozoan *Paramecium caudatum*, a natural prey, as a vehicle. We show that AIEC more robustly colonize the zebrafish gut and are cleared slower than non-pathogenic *E. coli*. In addition, DSS-induced enterocolitis increases bacterial burden and decreases bacterial clearance in the larval gut. We benchmark our model against existing rodent models using two mutants deficient in the known AIEC virulence factors FimH and IbeA, which have virulence defects in both rodent and the larval zebrafish model. Finally, we show that AIEC colonization exacerbates DSS-induced enterocolitis and prevents recovery from inflammation-induced damage. In conclusion, we established a high-throughput, genetically tractable model to study AIEC-host interactions in the context of pre-existing inflammation.

**IMPORTANCE:**

Although inflammatory bowel diseases are on the rise, what factors influence IBD risk and severity, and the underlying mechanisms remain to be fully understood. Although host genetics, microbiome, and environmental factors have all been shown to correlate with the development of IBD, cause and effect are difficult to disentangle in this context. For example, AIEC is a known pathobiont found in IBD patients, but it remains unclear if gut inflammation during IBD facilitates colonization with AIEC, or if AIEC colonization makes the host more susceptible to pro-inflammatory stimuli. It is critical to understand the mechanisms that contribute to AIEC infections in a susceptible host in order to develop successful therapeutics. Here, we show that the larval zebrafish model recapitulates key features of AIEC infections in other animal models and can be utilized to address these gaps in knowledge.

## INTRODUCTION

Inflammatory bowel disease (IBD) is a broad term for a range of chronic gastrointestinal disorders, including Crohn’s disease (CD) and ulcerative colitis (UC). IBD is prevalent in industrialized nations, and the number of cases in low-incidence areas is expected to keep rising (1, 2). Although the exact cause of IBD is unknown, host genetics, environmental factors, and the gut microbiota are all known disease modifiers (2).

Adherent-invasive *Escherichia coli* (AIEC) is a bacterial pathobiont that colonizes the gut of both healthy subjects and IBD patients but has a higher incidence in the diseased mucosae of patients with CD (21%–63%) and UC (0%–35.7%), (3–5). AIEC adhere to and invade intestinal epithelial cells, and survive inside macrophages without inducing host cell death *in vitro*, but how exactly they contribute to IBD is not well understood (6). It is thought that AIEC modify the pro-inflammatory environment, or inflammation facilitates AIEC colonization because they are often isolated from lesions in patients with chronic CD as opposed to those in remission (3, 7).

Current animal models of AIEC include mice that express the human carcinoembryonic antigen-related cell adhesion molecule 6 (CEACAM6) receptor (CEABAC10 mice), conventional mice treated with broad-spectrum antibiotics, mice treated with colitis-inducing agents [dextran sulfate sodium (DSS) and 2,4,6-trinitro-benzene sulfonic acid], and mice that are genetically susceptible to spontaneous colitis (8, 9). Although mice are powerful model organisms, they have some limitations including expensive care, long development periods, and laborious genetic manipulation. Furthermore, the scope of intravital imaging, particularly across multiple time points, in mice is limited, and consequently, observation of bacterial invasion, bacteria-phagocyte interactions, and pathophysiological changes often require euthanasia. To address the abovementioned gaps in knowledge, an animal model is needed that allows dynamic, high-throughput analyses and imaging of bacteria-host cell interactions in live animals.

The larval zebrafish (*Danio rerio*) has emerged as a powerful tool to study bacterial gastrointestinal infections because the gastrointestinal tract of larval zebrafish is physiologically and functionally similar to the human intestine (10–12). Other benefits that make zebrafish an effective high-throughput model organism include high fecundity, genetic tractability, and optical transparency throughout early development (10). Recent studies have used larval zebrafish to identify novel anti-inflammatory therapeutics for IBD and have shown that zebrafish harbor several known IBD susceptibility genes (13–15). A recently developed adult zebrafish model demonstrated the ability of a probiotic *E. coli* strain to decrease AIEC colonization (16).

Here, we set out to establish a model that combines a drug-inducible DSS enterocolitis model (17) and food-borne colonization with AIEC, to investigate the interplay between host inflammation and AIEC colonization. We use the protozoan *Paramecium caudatum*, a natural prey of larval zebrafish, as a vehicle to deliver AIEC to the larval intestine, as we have previously described for other enteric pathogens (18, 19). We benchmark this model using mutants of two AIEC virulence factors, FimH and IbeA, with known virulence deficiencies in rodent models (20, 21). We show that deletion of a type 1 pilus gene (*fimH*) and the gene encoding the invasion of the brain endothelium protein A (*ibeA*) results in decreased AIEC burden, neutrophil recruitment, and epithelial damage. We also show that IbeA contributes to AIEC invasion *in vivo*. Finally, we demonstrate that colonization with AIEC hampers recovery of the intestinal epithelium from damages sustained through underlying inflammation.

## RESULTS

### Adherent-invasive *E. coli* LF82 colonizes the larval zebrafish intestine better than non-pathogenic *E. coli* MG1655

We have previously established the protozoan *P. caudatum*, a natural prey of larval zebrafish, as a vehicle for zebrafish infection with enteric pathogens and non-pathogenic *E. coli* (18, 19, 22, 23). Internalization of bacteria by *P. caudatum* and subsequent ingestion of bacteria-loaded paramecia by larvae allow for the delivery of a higher bacterial dosage compared to bath immersion, which is commonly used in other zebrafish infection models including the adult zebrafish AIEC model (16, 19, 24). The uptake of bacteria-loaded paramecia by larvae is followed by digestion of the paramecia in the anterior gut and the subsequent release of bacteria into the intestine within 30 minutes of ingestion (19).

**Fig 1:**
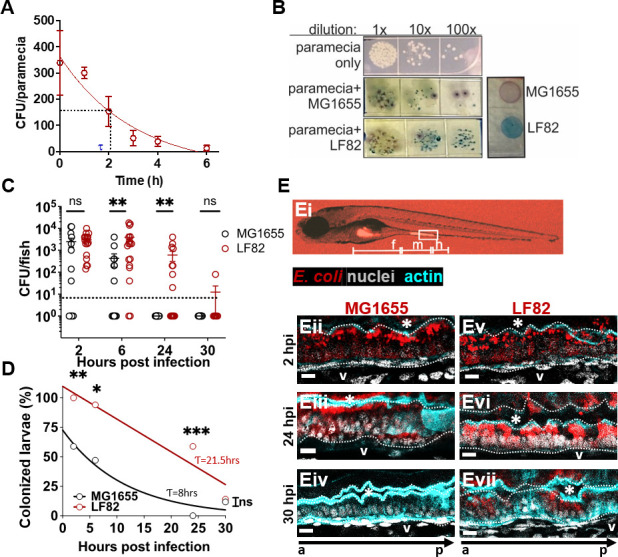
AIEC LF82 colonizes the larval zebrafish intestine better than MG1655. (**A**) AIEC-loaded paramecia sampled from 0 to 6 hours post incubation, and CFU/paramecia was calculated. AIEC half-life (τ) in paramecia is 2.1 hours. Data are means ± SEM, *n* = 3. (**B**) Bacterial colonies from tissue homogenates grown on CHROMagar O157. The zebrafish microbiota (white colonies) can be distinguished from AIEC LF82 (dark blue colonies) and *E. coli* MG1655 (mauve colonies). (**C**) Quantification of LF82 and MG1655 CFUs/fish. Fish with CFU below the detection limit (10 CFU/fish, dashed line) were annotated as 1 CFU. Data are from individual fish (*n* = 14) and means ± SEM. (**D**) Colonized larvae (%) are the percentage of fish with a burden above the detection limit; *n* = 14. Non-linear regression, first-order decay, ROUT outlier test with *Q* = 0.2%, paired *t*-test and Wilcoxon test. *, *P* ≤ 0.05; **, *P* ≤ 0.01; ***, *P* ≤ 0.001, ns, not significant. (**E**) Images of larvae colonized with *E. coli* (red), (Ei) whole larva at 10× magnification with intestinal segments (foregut (F), midgut (M), hindgut (H)) marked. (Eii–vii) Sagittal views of the midgut of larvae colonized with MG1655 (Eii–iv) and LF82 (Ev–vii) at 2, 24, and 30 hpi. The dotted white line outlines the intestinal epithelium and separates it from the lumen, indicated by *, and the blood vessel below the basement membrane (V). a to p marks anterior to posterior orientation; Scale bars = 100 um, *E. coli* (red), phalloidin (cyan, cell outline), nuclei (4′,6-diamidino-2-phenylindole, white), images are representative of *n* = 3.

Initially, we investigated the degradation and half-life of AIEC strain LF82 following uptake into *P. caudatum* vacuoles. The uptake of AIEC by paramecia occurred rapidly, with an average burden of 339 colony-forming units (CFUs) per paramecia quantified minutes after the introduction of AIEC (Fig. 1A). This is in accordance with other studies that show paramecia engulf their target within seconds to minutes (19, 25). The half-life τ of AIEC LF82 inside of paramecia was approximately 2.1 hours (Fig. 1A) and was used to determine the bacterial dosage consumed by larvae following a 2-hour incubation with AIEC-loaded paramecia, as done previously (18). The half-life of AIEC in paramecia was similar to that reported for EHEC (19), so bacteria and *P. caudatum* concentrations were kept as described previously.

Next, we quantified the bacterial burden of AIEC LF82 in zebrafish over 30 hpi, and used the non-pathogenic *E. coli* strain MG1655 as a control. Tissues from infected fish were homogenized and plated on CHROMagar O157, which allowed us to distinguish AIEC LF82 (steel-blue colonies) from *E. coli* strain MG1655 (mauve), and the larva’s endogenous microbiota (white, Fig. 1B). Following food-borne delivery, AIEC and MG1655 were taken up by the larvae at similar concentrations (Fig. 1C, 2 hpi). At later time points (6–24 hpi), AIEC formed a significantly higher burden within fish than non-pathogenic *E. coli* MG1655 (Fig. 1C). The number of fish with a bacterial burden below the detection limit increased after 6 hpi, and by 24 hpi, no MG1655 was detected in any of the fish (Fig. 1C). To get a better representation of the difference in bacterial clearance between LF82 and MG1655, we quantified the percentage of fish that contained a burden of AIEC or MG1655 above the detection limit (≥10 CFU/fish). Although both strains were cleared from the intestine over time, AIEC LF82 (half-life 21.5 hours) was cleared slower than non-pathogenic *E. coli* MG1655 (half-life of 8 hours), (Fig. 1D). Neither colonization with MG1655 nor LF82 caused any mortality throughout the experimental time course (Fig. S1).

We visualized the site of bacterial colonization within the zebrafish larvae using fluorescent AIEC LF82::mCherry and MG1655::mCherry strains. At 2 hpi, both strains were visible in the foregut lumen and attached to the midgut epithelium (Fig. 1Ei). The localization of *E. coli* relative to the intestinal epithelium was assessed using a nuclear stain and phalloidin to outline the epithelium (Fig. 1Eii through Evii). High-resolution fluorescence microscopy of the midgut revealed that individual AIEC and MG1655 cells localized both along the epithelial surface and inside the epithelium (Fig. 1Eii and Ev). By 24 hpi, luminal bacteria were no longer observed, and the burden of MG1655 had decreased (Fig. 1Eiii), while the LF82 burden had increased, with more invasion visible (Fig. 1Evi). At 30 hpi, MG1655 was no longer visible (Fig. 1Eiv), while AIEC LF82 was still observed within the epithelium (Fig. 1Evii). Taken together, these experiments showed that AIEC forms a higher burden, and its clearance from the larval gut is slower than for non-pathogenic *E. coli*, most likely due to invasion of the intestinal epithelium.

### Larval immersion in 0.5% DSS recapitulates key morphological and pro-inflammatory features of previously described DSS enterocolitis models

Although AIEC is found in gastrointestinal biopsies from healthy hosts, it is more prevalent in hosts experiencing pre-existing inflammation, such as patients suffering from IBD (26–28).

To address whether pre-existing inflammation affects bacterial colonization and clearance, we expanded the larval model to include drug-induced intestinal inflammation. DSS is a chemical agent that induces enterocolitis in larval zebrafish, with pathological features similar to those of chronic colitis in rodents (17, 29–31).

**Fig 2:**
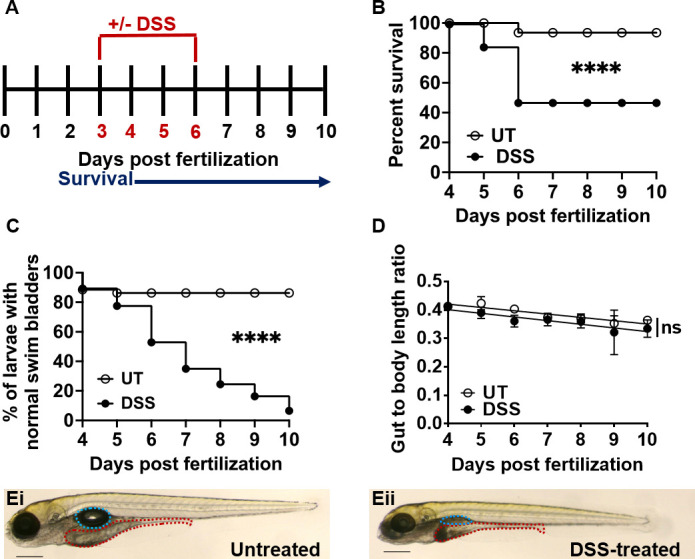
Larval zebrafish treated with 0.5% DSS have decreased survival and intestinal growth rates. (**A**) Schematic outlining timeline of DSS administration (red, 3–6 dpf) and survival experiments (blue, 1–7 days post exposure). (**B**) Survival of larvae administered 0.5% DSS (black circles) relative to untreated (UT) controls (empty circles). Data were analyzed using a Kaplan-Meier plot and Mantel-Cox test; ****, *P* ≤ 0.0001, *n* = 20. (**C**) Quantification of swim bladder defects in UT or DSS-treated larvae. Group differences were analyzed using Mantel-Cox test; ****, *P* ≤ 0.0001, *n* = 20. (**D**) Gut-to-body length ratio was analyzed by linear regression; ns = not statistically significant. Data are means ± SEM from *n* = 20. (**E**) Representative images of untreated (Ei) and DSS-treated (Eii) larvae at 6 dpf (3 days post DSS exposure), with the swim bladder (teal) and the intestine (red) outlined. Scale bar = 0.3 mm.

**Fig 3:**
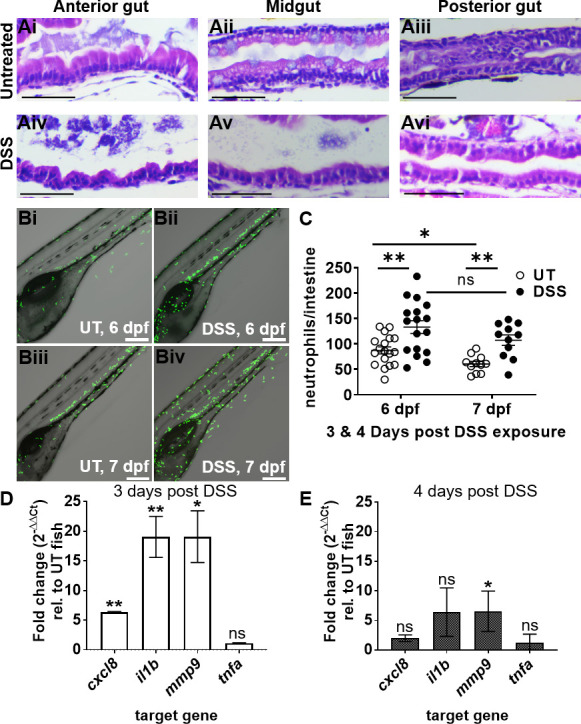
DSS causes intestinal epithelial damage and inflammation consistent with enterocolitis. (**A**) Representative hematoxylin- and eosin-stained longitudinal sections (*n* = 4) of the anterior, mid, and posterior intestine from untreated (Ai–iii) and DSS-treated (Aiv–vi) larvae at 6 dpf; scale bars = 50 µm. (**B**) Representative confocal images of live UT (i, iii) and DSS-treated (ii, iv) Tg(mpo::egfp) larvae at 6 (i-ii) and 7 (iii-iv) dpf; neutrophils (green); larvae were imaged for 18 hours (3–20 hpi). Scale bars = 200 µm. (**C**) Quantification of neutrophils in the intestine at 6 and 7 dpf (3 and 4 days post DSS treatment); unpaired two-tailed *t*-test, *n* ≥ 11. (**D**) qRT-PCR analyses of *cxcl8, il1b, mmp9,* and *tnfa* in DSS-treated larvae relative to untreated controls at 6 dpf and (**E**) 7 dpf; *n* = 3. Unpaired two-tailed *t*-test. Mean ± SEM, *, *P* ≤ 0.05; **, *P* ≤ 0.01; ns, not significant.

To replicate previously described DSS models, we tested different DSS concentrations and assessed larval survival, development, and inflammation (Fig. 2 and 3; Fig. S2). The goal was to find a DSS-dosing regimen that would induce a robust pro-inflammatory response without causing excessive mortality. Based on the experimental parameters previously described by Oehlers et al. (17, 29), we immersed larval zebrafish in E3 media containing 0.25%–0.75% DSS from 3 to 6 dpf, replacing the solution daily (Fig. 2A). Over the course of 4–10 dpf (7 days post DSS treatment), the percent survival of larvae administered 0.5% DSS decreased to 48% in comparison to untreated controls (Fig. 2B). The survival of DSS-treated and untreated larvae was similar at 4 and 5 dpf (1 and 2 days post treatment); however, changes in the survival rate were observed at 6 dpf (3 days post treatment) (Fig. 2B). We observed that larval survival stabilized 3 days after the DSS was removed, and no additional mortality was observed from 7 to 10 dpf. In comparison, larvae administered 0.25% DSS had a 100% survival rate, and those administered 0.75% DSS did not survive past 6 dpf (3 days post DSS exposure) (Fig. S2A). Consequently, we further assessed the development and inflammatory responses of larval fish treated with 0.5% DSS.

Prolonged treatment with 0.5% DSS led to abnormal swim bladder development over time (Fig. 2C and E) and slightly stunted the elongation of the larval gut and overall body length (Fig. 2D and E; Fig. S2B and C). Analysis of the gut to whole body ratio of untreated and DSS-treated larvae suggested that DSS did not disproportionally affect gut development and that shorter gut length was a consequence of overall shorter body length since there was no significant difference in slope between untreated and DSS larvae (Fig. 2D). Hematoxylin and eosin (H&E) staining and histology of paraffin embedded, sectioned larvae revealed normal morphology of the anterior, mid, and posterior gut of untreated larvae (Fig. 3A). The intestinal epithelium was intact, with intestinal folds visible in the anterior gut and mucus-producing goblet cells in the midgut epithelium (Fig. 3Ai through iii). In contrast, the epithelium was disrupted in DSS-treated larvae, with visible fraying, corrosion of intestinal folds, and epithelial detachment from the basement membrane in all three gut segments (Fig. 3Aiv through vi).

Next, we studied phagocyte recruitment during DSS-induced intestinal inflammation using transgenic larvae containing fluorescent neutrophils [Tg(*mpo::egfp*)] and macrophages [Tg(*mpeg1::egfp*)], respectively. Neutrophils are used as a readout for intestinal inflammation because they are the first responders to injuries and infections (32–35). Macrophages are also involved in the tissue repair and clearance of spent neutrophils but appear at later time points (36). Live imaging of 6–7 dpf larvae allowed us to quantitate the number of neutrophils infiltrating the intestine. We observed that neutrophil recruitment to the intestine was significantly increased in DSS-treated vs untreated larvae at both 6 and 7 dpf (corresponding to 3 and 4 days of DSS treatment, respectively) (Fig. 3B and C). In contrast, there was no change in the number of macrophages infiltrating the gut in untreated vs DSS-treated fish (Fig. S3). To further evaluate pro-inflammatory signaling, we quantified the expression of the key pro-inflammatory markers interleukin 8 (*cxcl8-l1*), interleukin-1-β (*il1b*), matrix metallopeptidase 9 (*mmp9*), and tumor necrosis factor-alpha (*tnfa*) at 6 and 7 dpf (corresponding to 3 and 4 days of DSS treatment, respectively). At 6 dpf, the relative expression of *cxcl8*, *il1b*, and *mmp9* was significantly increased in DSS-treated larvae compared to untreated controls, whereas *tnfa* expression remained constant (Fig. 3D). By 7 dpf, the relative expression of *cxcl8*, *il1b,* and *tnfa* was similar in DSS-treated and untreated fish, whereas *mmp9* expression remained elevated (Fig. 3E). Taken together, these data recapitulate key morphological and pro-inflammatory features of previously described DSS enterocolitis models and support our methodology of immersing larvae in 0.5% DSS from 3 to 6 dpf to induce inflammation prior to introducing bacteria.

### Pre-existing intestinal inflammation enhances AIEC LF82 colonization and invasion of the gut epithelium

**Fig 4:**
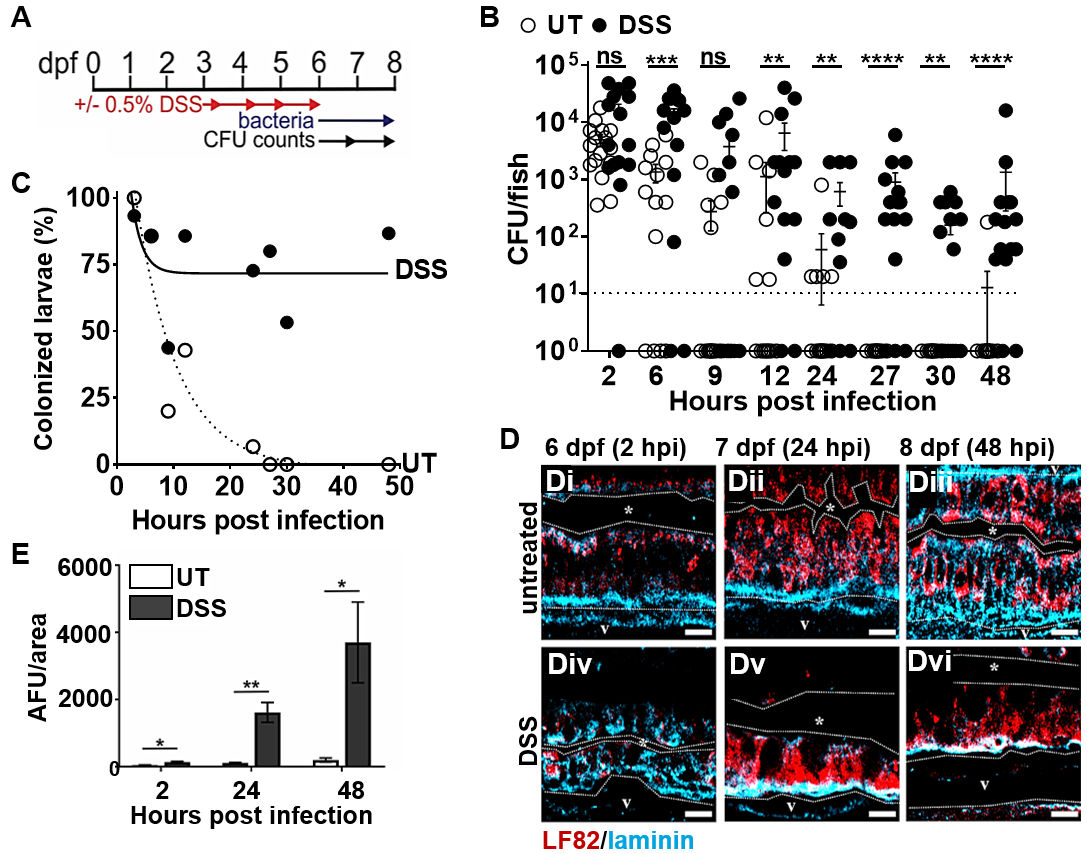
Pre-existing intestinal inflammation enhances the colonization and invasion of AIEC LF82. (**A**) Timeline of DSS administration, infection of larvae, and sampling for CFU counts. (**B**) Quantification of LF82 CFUs per larvae with and without DSS treatment, *n* ≥17; fish with CFU below the detection limit (10 CFU/fish, dashed line) were annotated as 1 CFU. (**C**) Colonized larvae (%) are percentage of fish with a burden of AIEC above the detection limit; non-linear regression first-order decay, ROUT outlier test with *Q* = 0.2%. (**D**) LF82 (red) in the mid-intestine of UT (Di–iii) and DSS-treated (Div–vi) larvae relative to the basement membrane (blue) from 2 to 48 hpi or 6–8 dpf. The dotted white line outlines the intestinal epithelium and separates it from the lumen, indicated by *, and the blood vessel below the basement membrane (V). Scale bars = 10 µm. (**E**) Quantification of red fluorescence intensity (AFU) (representing AIEC) in the vasculature (V) at 2, 24, and 48 hpi, *n* = 6; *, *P* ≤ 0.05; **, *P* ≤ 0.01; ***, *P* ≤ 0.001; ****, *P* ≤ 0.0001; ns, not statistically significant.

Next, we asked whether DSS-induced intestinal inflammation would affect the outcome of subsequent colonization by AIEC (or the non-pathogenic MG1655 strain as a control). Following the 3-day DSS exposure, we introduced AIEC LF82 to larval zebrafish via food-borne infection (Fig. 4A). Larvae that had become moribund or had a deflated swim bladder following the initial DSS treatment were excluded from subsequent infection experiments. At 2 hpi, the AIEC burden in DSS inflammation fish was similar to the AIEC burden in untreated fish (Fig. 4B). However, the burden of AIEC in DSS-treated larvae was higher than that of the untreated controls at 6 and 12–48 hpi (Fig. 4B). Furthermore, clearance of LF82 from DSS-treated larvae was significantly slower compared to untreated fish (Fig. 4C). Together, these data suggest that pre-existing inflammation enhances the burden and slows the clearance of LF82 in the intestine of larval zebrafish. These results are also in accordance with those of published murine studies that show that AIEC persists longer in mice with IBD compared to healthy controls (37–39).

To investigate whether pre-existing inflammation enhances bacterial colonization in general, or specifically for AIEC, the colonization patterns of MG1655 in DSS-treated larvae were also assessed. The burden of LF82 was significantly higher and bacterial clearance slower than that of MG1655 in DSS-treated fish at 2, 6, 24, and 48 hpi (Fig. S4A and B). These results demonstrate that pre-existing intestinal inflammation enhances the burden of both AIEC and non-pathogenic *E. coli* and that AIEC LF82 still colonized better and was cleared slower compared to non-pathogenic *E. coli* in fish with enterocolitis.

Intestinal inflammation damages the mucosal barrier and enhances intestinal permeability, allowing for increased bacterial invasion (17, 40, 41). Therefore, we asked whether pre-existing intestinal inflammation would affect AIEC invasion in our model. DSS-treated and untreated larvae were infected with LF82, euthanized at 2, 24, and 48 hpi, and laminin and 4′,6-diamidino-2-phenylindole (DAPI) stained to assess the localization of LF82::mCherry relative to the intestinal lumen, epithelium, and underlying vasculature (Fig. 4D). At 2 hpi, LF82 cells were present within the epithelium of untreated and DSS-treated zebrafish and had begun to invade the underlying vasculature in DSS-treated but not in control fish (Fig. 4Di, Div, and E). At 24 hpi, individual bacterial cells remained visible in untreated larvae, whereas large bacterial aggregates were observed within the epithelium of DSS-treated fish (Fig. 4), and increased bacterial invasion of the underlying vasculature was observed in DSS fish but not untreated controls (Fig. 4E). By 48 hpi, the AIEC burden within the epithelium had lowered (Fig. 4Diii and Dvi), but invasion of the vasculature in DSS-treated fish was further elevated (Fig. 4E). Together, these data suggest that pre-existing enterocolitis facilitates bacterial colonization, slows bacterial clearance, and exacerbates invasion of the bloodstream by AIEC.

### AIEC LF82 exacerbates intestinal inflammation in DSS-treated larvae

**Fig 5:**
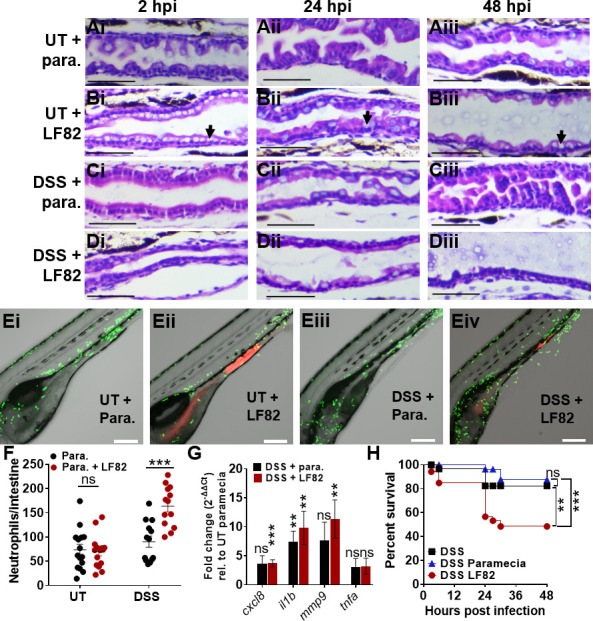
AIEC LF82 exacerbates intestinal inflammation in DSS-treated larvae. (**A–D**) H&E-stained longitudinal sections of the mid-intestine of larvae without (**A and B**) and with (**C and D**) prior DSS treatment, fed paramecia (para.) alone (**A, C**) or paramecia containing LF82 (**B, D**) at 2 (i), 24 (ii), and 48 (iii) hpi, *n* = 3. Black arrows point to goblet cells. Scale bars = 50 µm. (**E**) Representative confocal images of Tg(*mpo::egfp*) larvae fed paramecia only (i, iii) or LF82 (ii, iv) at 6 dpf. Larvae were imaged for 18 hours (3–20 hpi), neutrophils (green) and bacteria (red). Scale bars = 200 µm. (**F**) Quantification of neutrophils per intestine in UT- and DSS-treated fish fed with paramecia only (black) or para. containing LF82 (red); *n* ≥10. (**G**) qRT-PCR analyses of *cxcl8, il1b, mmp9,* and *tnfa* in DSS-treated (red) larvae infected with LF82- and DSS-treated larvae fed paramecia (black) relative to UT paramecia controls (onefold) at 6 dpf, *n* = 7. Unpaired two-tailed *t*-test. Mean ± SEM. (**H**) Survival of DSS-treated larvae that were uninfected (black), fed paramecia control (blue), or para. containing AIEC (red). *N* = 17. Kaplan-Meier and Mantel-Cox test, followed by a Bonferroni correction test. **, *P* ≤ 0.01; ***, *P* ≤ 0.001; ns, not statistically significant.

Murine studies show that colonization with AIEC LF82 exacerbates intestinal inflammation in DSS-treated animals and causes an immunopathology similar to that observed in IBD patients (39, 42, 43). Thus, we investigated whether AIEC could exacerbate inflammation in larvae with pre-existing DSS enterocolitis. Untreated and DSS-treated larvae fed the paramecia vehicle only (uninfected) were used as controls and compared to AIEC-infected fish (Fig. 5). The midgut of untreated fish colonized with LF82 contained more mucus-secreting goblet cells at 2, 24, and 48 hpi compared to control fish (Fig. 5A vs B, cells containing clear/light blue mucous droplets) (44). More goblet cells were also observed in the posterior gut of untreated larvae infected with LF82 from 2 to 48 hpi (Fig. S5F).

Following DSS treatment, we observed corrosion of intestinal folds in the midgut ((Fig. 5Ci) and anterior gut (Fig. S5C) at 6 dpf (corresponding to 3 days post-DSS treatment). In uninfected fish, these folds were partially restored at 7 and 8 dpf (1–2 days after DSS treatment had stopped, Fig. 5Cii through Ciii; Fig. S5C), suggesting that the intestinal epithelium can recover from damage caused by enterocolitis.

In contrast, DSS-treated larvae infected with LF82 were unable to fully recover from enterocolitis by 48 hpi since the anterior and midgut did not recover the original intestinal fold architecture and exhibited a thinner epithelial cell layer compared to DSS-treated larvae that were not infected (Fig. 5D vs C; Fig. S5D vs C). LF82 colonization did not disrupt intestinal folds in the absence of DSS inflammation (Fig. 5B; Fig. S5B). Together, these data suggest that AIEC LF82 alters the architecture of the intestine of larvae; in untreated fish. LF82 increases goblet cell number; and in DSS-exposed fish, it prevents epithelial healing. The increased presence of mucin-producing goblet cells may indicate a host-defense response to fight off bacterial infections, whereas flattening of the intestinal villi may be due to inflammation (45).

To further examine the effect of LF82 on inflammation, neutrophil recruitment was assessed, and induction of inflammatory markers was quantified using qRT-PCR. In untreated fish, AIEC colonization did not affect neutrophil recruitment to the gut (Fig. 5E and F). Similarly, in uninfected fish, neutrophil recruitment to the intestine was unchanged following DSS treatment (Fig. 5E and F). In contrast, DSS treatment and subsequent AIEC colonization had an additive effect and increased neutrophil recruitment (Fig. 5E and F). Macrophage recruitment to the intestine was not significantly affected by either DSS treatment or AIEC infection (Fig. S6). Expression of inflammatory markers *cxcl8-l1, il1b*, and *mmp9* was slightly elevated following DSS treatment alone and significantly increased in DSS-treated fish colonized with AIEC (Fig. 5G). Comparison of inflammatory marker expression following AIEC colonization of untreated or DSS-treated fish further showed that DSS treatment and AIEC infection have an additive effect on pro-inflammatory signaling (Fig. S7).

The observed increase in epithelial damage and pro-inflammatory response following LF82 infection in DSS-treated fish may contribute to the increase in mortality of DSS-treated larvae infected with AIEC LF82, relative to DSS alone or DSS larvae fed paramecia only (Fig. 5H). Together, these data suggest that while AIEC colonization in healthy fish causes little epithelial damage and inflammation, it exacerbates inflammation and tissue damage in hosts with pre-existing enterocolitis.

### FimH and IbeA contribute to AIEC virulence in larval zebrafish

Next, we investigated whether the larval zebrafish model is suitable for the characterization and/or identification of virulence factors involved in *in vivo* infections by characterizing the phenotypes of two known AIEC virulence factors, FimH and IbeA, as a benchmark. FimH is the terminal subunit of type I pili and binds collagen type I and type IV, laminin, fibronectin, and mannosylated glycoproteins (46). FimH of AIEC LF82 adheres to the human CEACAM6 receptor that is abnormally expressed in the ileum of CD patients and expressed in transgenic CEABAC10 mice (8, 21). The presence of CEACAM6 receptors in a host is thought to promote colonization with AIEC and to indirectly contribute to intestinal inflammation since binding of AIEC to CEACAM6 through FimH triggers intestinal inflammation in CEABAC10 mice (47). IbeA is an invasin and outer membrane protein conserved in the *E. coli* phylogenetic group B2, which includes avian pathogenic *E. coli*, newborn meningitis-causing *E. coli*, and AIEC strains NRG857C and LF82 (20). BLAST analyses show that the IbeA protein in these pathogenic *E. coli* strains is 100% identical (data not shown). IbeA binds to vimentin found in macrophages, fibroblasts, and endothelial cells, and mediates the invasion of Caco-2 and M-like cells by AIEC strain NRG857c (20).

To investigate whether FimH and IbeA play a role in colonization and invasion of AIEC LF82 in zebrafish larvae, these genes were deleted from the parent strain and complemented by inserting *fimH* or *ibeA* with their endogenous promoters into the chromosome. Deletion and complementation of either gene did not affect the overall growth of AIEC LF82 (Fig. S8). There were no fortuitous mutations identified in the deletion and complement strains, which were subjected to whole-genome sequencing.

**Fig 6:**
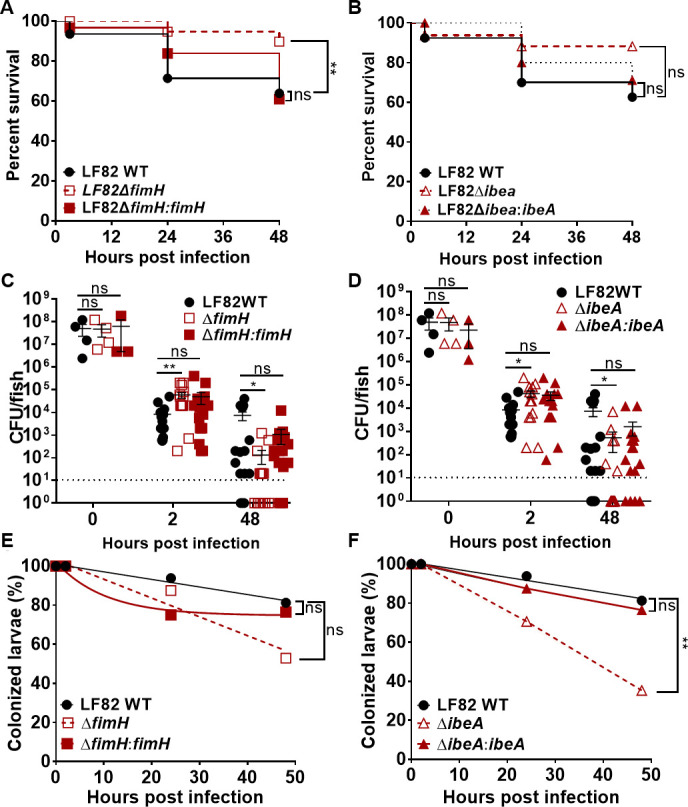
Effects of *fimH* and *ibeA* deletion on larval survival, bacterial burden, and bacterial clearance. Survival of larvae infected with (**A**) LF82 wild-type (WT), LF82Δ*fimH*, LF82Δ*fimH:fimH* or (**B**) LF82, LF82Δ*ibeA*, LF82Δ*ibeA:ibeA* at 2, 24, and 48 hpi. Kaplan-Meier and Mantel-Cox test, followed by a Bonferroni correction test, *n* = 20. Quantification of bacterial burden and clearance of (**C, E**) LF82, LF82Δ*fimH*, LF82Δ*fimH:fimH*, or (**D, F**) LF82Δ*ibeA*, and LF82Δ*ibeA:ibeA* in DSS-treated larvae from 2 to 48 hpi. Fish with CFU below the detection limit (10 CFU/fish, dashed line) were annotated as 1 CFU. Significance of difference in burden was analyzed using a Kruskal-Wallis test, *n* ≥16. Bacterial clearance (percent of fish with a burden of AIEC above the detection limit) was analyzed using a log-rank test. Non-linear regression, first-order decay graph used to model bacterial clearance. *, *P* ≤ 0.05; **, *P* ≤ 0.01; ns, not significant; Experiments for the WT and both mutant strains were performed in parallel, and thus, data for the WT strain are duplicated between panels A and B, C and D, and E and F, respectively.

**Fig 7:**
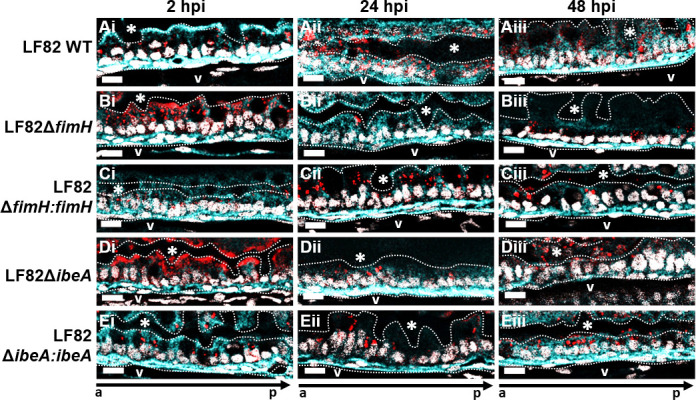
Deletion of *ibeA*, but not *fimH*, results in aggregation and retention of AIEC LF82 on the epithelial surface. Representative sections (*n* = 3) of the mid-intestine of larvae infected with (**A**) LF82 WT, (**B**) *LF82ΔfimH*, (**C**) *LF82ΔfimH:fimH*, (**D**) LF82Δi*beA:ibeA*, or (**E**) LF82Δi*beA:ibeA* at (i) 2, (ii) 24, and (iii) 48 hpi. AIEC LF82 (red), laminin (cell surface, cyan), nuclei (DAPI, white). The dotted white line outlines the intestinal epithelium and separates it from the lumen, indicated by *, and the blood vessel below the basement membrane (V). a to p marks anterior to posterior orientation; Scale bars represent 10 µm.

Deletion of *fimH* but not of *ibeA* significantly increased larval survival, and the defect was restored in the LF82Δ*fimH:fimH* complementation strain (Fig. 6A and B). The *fimH* and *ibeA* deletion and complementation strains were taken up into the larval gut at similar levels than the wild-type strain (Fig. 6C and D, 0 hpi). Interestingly, deletion of either *fimH* or *ibeA* initially increased AIEC colonization but led to a colonization defect at 48 hpi. Complementation of *fimH* and *ibeA* restored wild-type colonization levels (Fig. 6C and D). Bacterial clearance was unaffected by *fimH* deletion (Fig. 6E) but decreased upon deletion of *ibeA* (Fig. 6F). Next, we asked whether the deletion of *fimH* or *ibeA* affected the invasion of the epithelium by AIEC. Infected larvae were euthanized, fixed, and stained with anti-laminin and DAPI to visualize the localization of LF82Δ*fimH:mcherry* and LF82Δ*ibeA:mcherry* and complementation strains over the course of 48 hpi. Deletion of either *fimH* or *ibeA* caused a transient increase in bacterial burden at 2 hpi (Fig. 7A through Ei), followed by significantly decreased colonization at 24–48 hpi (Fig. 7A through Eii and Eiii) consistent with the CFU burden data (Fig. 6C and D). Interestingly, while the *fimH* mutant was still able to invade the epithelium, the *ibeA* mutant mainly colonized and formed aggregates at the epithelial surface (Fig. 7D). Complementation of *fimH* and *ibeA* restored wild-type adherence and invasion (Fig. 7C and E). These data suggest that FimH and IbeA both contribute to aspects of pathogenesis but play distinct roles in bacterial adherence and invasion.

### FimH and IbeA elicit a pro-inflammatory response during AIEC colonization and prevent epithelial recovery from enterocolitis

**Fig 8:**
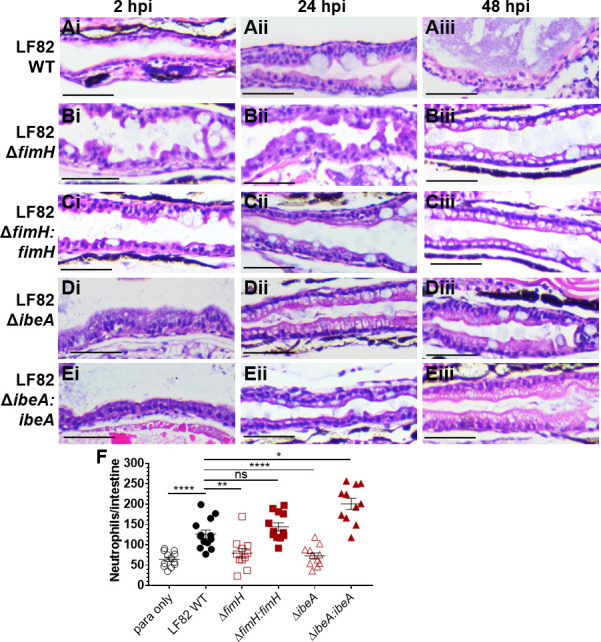
Deletion of *fimH* and *ibeA* in AIEC LF82 results in decreased tissue damage and neutrophil recruitment to the intestine compared to LF82. H&E longitudinal sections of the mid-intestine of larvae infected with (**A**) LF82 WT, (**B**) LF82Δ*fimH,* (**C**) LF82Δ*fimH:fimH,* (**D**) LF82Δ*ibeA*, and (**E**) LF82Δ*ibeA:ibeA* at 2 (i), 24 (ii), and 48 (iii) hpi. Representative images for *n* = 3. Scale bars = 50 µm. (**F**) Quantification of neutrophils per intestine for DSS-treated fish infected with abovementioned LF82 strains or paramecia-only control. Kruskal-Wallis test. *n* ≥11. *, *P* ≤ 0.05; **, *P* ≤ 0.001; ****P ≤ 0.0001; ns, not significant.

Since both FimH and IbeA are bacterial surface proteins, we next asked if they contribute to the pro-inflammatory response to AIEC colonization in DSS-treated fish. Histology of midgut sections from infected DSS-treated fish showed that colonization with wild-type or complementation strains prevented recovery from DSS intestinal inflammation, and corrosion of intestinal folds persisted even 2 days after DSS treatment had been discontinued (Fig. 8A, 24–48 hpi). In contrast, healthy epithelial morphology was restored following infection with either *fimH* or *ibeA* deletion strains (Fig. 8B and D). Lastly, we studied how FimH and IbeA contribute to AIEC immunogenicity, by quantifying neutrophil recruitment to the gut. Fish infected with LF82 WT recruited more neutrophils to the intestine compared to either uninfected, paramecia-fed fish, or fish harboring LF82Δ*fimH* and LF82Δ*ibeA* (Fig. 8F). Complementation of *fimH* resulted in increased neutrophil recruitment similar to or in the case of ibeA, more neutrophil recruitment than wild-type infection. Taken together, these data suggest that both FimH and IbeA contribute to pro-inflammatory signaling in response to AIEC infection and also contribute to attenuation of epithelial recovery in DSS enterocolitis fish.

## DISCUSSION

In this study, we establish larval zebrafish as a model to study the interplay between host inflammatory responses and AIEC colonization. During the initial hours post infection, AIEC is observed colonizing the foregut and the midgut; however, over the course of infection, AIEC shows a preference for colonizing the midgut of larvae, similar to EHEC (19). This region of the intestine contains absorptive enterocytes, mucin-secreting goblet cells, and M-like cells, all of which are also found in the mammalian small intestine (48). Accordingly, AIEC predominantly colonizes the small intestine rather than the colon of IBD patients (3, 49–51).

By combining a previously published DSS enterocolitis model (17) with food-borne AIEC infection, we were able to analyze host-microbe interactions in a dynamic fashion, using intravital and high-resolution imaging of live and euthanized larvae. AIEC colonizes the larval gut better than non-pathogenic *E. coli*, which is in accordance with published murine studies (37). To date, it is unknown whether colonization with AIEC in a susceptible host triggers the onset of intestinal inflammation, or if pre-existing inflammation creates a favorable environment for the AIEC pathotype. Our data suggest that AIEC colonizes and promotes inflammation in healthy hosts but is particularly adapted to colonize hosts with ongoing enterocolitis. While uninfected hosts recover from intestinal inflammation after removal of pro-inflammatory stimuli (here, DSS), tissue repair and healing are impaired in hosts colonized with AIEC.

There are a few differences between rodent and zebrafish models of AIEC. Although both mice and larval zebrafish have an endogenous microbiota, the rodent microbiome renders mice highly colonization resistant, and AIEC models involve antibiotic treatment to remove much of the endogenous microbiome to allow for bacterial colonization. In contrast, larval zebrafish do not need to be treated with antibiotics to remove the endogenous microbiome, and a single dose of 10^4^–10^5^ CFUs of AIEC consumed through food-borne infection is sufficient to promote bacterial colonization. Mouse models are usually challenged with 10^8^–10^9^ CFUs of AIEC through oral gavage daily for 3 or 15 days, making them more labor intensive (8, 38, 52). The existing zebrafish model of AIEC infection only requires bath immersion, but adult zebrafish are needed to achieve robust colonization (16). In larval zebrafish, we found bath immersion ineffective in establishing AIEC colonization. We found that AIEC colonization causes increased mortality in DSS enterocolitis fish, compared to unfed or paramecia-fed DSS-treated fish (Fig. 5H). This is consistent with mouse studies, where AIEC LF82, but not *E. coli* strain K 12, decreases the survival of CEBAC10 and DSS-treated mice (8).

AIEC LF82 exacerbates intestinal inflammation in hosts with pre-existing inflammation. This is supported by an increase in neutrophil recruitment to the intestine, the inability of the mid-intestine to heal while colonized with AIEC, and the increased expression of pro-inflammatory markers *cxcl8-l1, il1b*, and *mmp9*. Cxcl8 is primarily associated with the activation and mobilization of neutrophils, whereas Tnfα and Il-1β are involved in signaling pathways that regulate apoptosis and cell survival (53). Mmp9 degrades the extracellular matrix during inflammation and through this process activates cytokines that mediate tissue and wound healing (54); however, its activation can also contribute to intestinal damage during IBD (55).

To investigate whether *fimH* and *ibeA* are important for the colonization of AIEC in the zebrafish intestine, these two genes were deleted from the parent strain. Both *fimH* and *ibeA* have been extensively characterized *in vivo* and *in vitro*, and thus we used them to benchmark our model against published *in vivo* and *in vitro* AIEC models. Deletion of *fimH* significantly decreased LF82 colonization in two different mouse models that express mammalian CEACAM6 in the intestine (8, 56). Deletion of *ibeA* did not impact the burden of AIEC strain NRG857c in mice, although it did contribute to invasion and intracellular survival *in vitro* (20). In the larval zebrafish model, deletion of either *fimH* or *ibeA* transiently caused a higher bacterial burden early during infection but decreased bacterial burden at later time points (Fig. 6 and 7). It is possible that LF82 expresses additional virulence factors involved in adhesion, including OmpA, OmpC, long polar fimbriae, and the lipoprotein NlpI (57–61). Alternatively, the transient increase in burden could be due to an altered immune response since both FimH and IbeA are involved in neutrophil recruitment and pro-inflammatory signaling in our model (Fig. 8). It is possible that *fimH* or *ibeA* deletion causes a defect in bacterial clearance early during infection, and adhesion and invasion defects during later time points. In addition to their immunogenicity, FimH and IbeA both played a role in sustaining epithelial damage and prevention of healing in DSS-treated fish. It is likely that their role in pro-inflammatory signaling and blocking tissue recovery is linked. Our findings are in-line with other studies showing decreased epithelial corrosion in mice infected with *fimH* or *ibeA* mutants (8, 20).

Recently published work established adult zebrafish as a model of AIEC infection and showed that adult zebrafish produce S100A-10b, a calprotectin homolog, in response to intestinal inflammation caused by LF82 (16). This is in-line with our observation that AIEC induce inflammation in zebrafish. The decision to use adult or larval zebrafish to study AIEC depends on the type of readouts required to address a question of interest. In contrast to larvae, adult zebrafish are not transparent, which hinders dynamic imaging of single cells. However, adult fish have a functional adaptive immune system, which allows studies on this aspect of host-microbe interactions.

The reason why AIEC colonizes hosts with pre-existing inflammation more efficiently than healthy fish is not well understood, but there are several potential explanations. First, DSS damages the intestinal barrier and facilitates the adhesion and invasion of AIEC, which results in bacterial localization closer to the epithelial basement membrane (Fig. 4Dvi). As a result, the bacteria are farther away from the lumen and fail to be cleared out by peristaltic contractions (62). Within the basement membrane, fibronectin, collagen types IV, VII, and XVIII, and laminin are abundant, and these host proteins are all known to bind several bacterial adhesins (63). A second reason may be that DSS changes the composition of the intestinal microbiota that may otherwise limit AIEC colonization. Studies show that the administration of the colitis inducing drug 2,4,6-trinitro-benzene sulfonic acid to larval zebrafish changes the proportion of species belonging to the Proteobacteria and Firmicutes phyla (64). Third, intestinal inflammation may cause the overexpression of a receptor important for binding of AIEC. *In vitro* studies suggest that AIEC can increase the expression of host adhesin receptors. For example, the binding of LF82 through FimH to CEACAM6 induces blebbing of apoptotic cell-derived membranous vesicles, which exposes oligomannosidic glycans that serve as AIEC-binding sites (65). Moreover, the expression of CEACAM6 is increased by TNFα and IFN-γ following AIEC infections (66). CECAM receptors are cell-surface glycoproteins expressed in epithelial, endothelial, and myeloid cells (8, 67). To date, 12 human CEACAM receptors have been identified and fully characterized (67). In contrast, 10 putative CEACAM receptors have been identified in the zebrafish genome, and only 1 CEACAM protein (CEACAMz1) has been characterized. CEACAMz1 is predominantly expressed in gills and, to a lesser extent, in the intestine (68). Mammalian CEACAM6 is also expressed in the alveolar and airway epithelial cells of the lungs under homeostatic conditions and is highly expressed in the gut only during intestinal disease (69). Furthermore, larval zebrafish express a CEACAM6-like protein (encoded by the *zgc:198329* gene) in the intestine that is 29% identical to human CEACAM6 (70). Future studies are required to investigate to what extent CEACAM proteins are involved in the binding of AIEC in the zebrafish intestine.

Whether or not AIEC contain specific molecular signatures is not currently known, but it has been proposed that there are undiscovered AIEC-specific genes that are not commonly found in non-pathogenic *E. coli* strains that are yet to be identified (71). It has been argued that a plausible reason that such molecular markers have not yet been discovered arises from the limitations of currently used infection models and *in vitro* models to classify *E. coli* strains as AIEC (72, 73). Currently, classification of strains as AIEC relay on *in vitro* assays to quantify adhesion, invasion, and replication inside of infected cells since there are no widely conserved genetic features, such as the LEE pathogenicity islands in EHEC/EPEC, or certain toxins, such as in the case of STEC (Shiga toxins) and ETEC (LT/ST enterotoxins). However, it is plausible that there may be genes essential for AIEC *in vivo* colonization that are not expressed in a simplified *in vitro* model or are disproportionately important in facilitating colonization only in hosts with pre-existing inflammation. Comparative transcriptomic studies show that the pathogenicity of AIEC changes when AIEC cells are grown *in vitro* and in the presence of host factors (49, 73, 74). These are hypotheses that may be addressed using transposon mutagenesis and high-throughput assays in larval zebrafish. We also propose that larval zebrafish may facilitate the screening of drugs that target AIEC. Positive results regarding microbial virulence factors, host factors contributing to disease progression, and initial drug candidates in larval zebrafish may then be further evaluated in mammals. We expect this to present a cost-effective way to identify novel genes that link AIEC with the development or progression of IBD.

## MATERIALS AND METHODS

### Zebrafish maintenance and breeding

The zebrafish lines used in this study were wild-type (AB) and transgenic lines Tg(*mpo::egfp*) (75) and Tg(*mpeg1:egfp*) (76), which express EGFP in neutrophils and macrophages, respectively. Adult fish were kept in a recirculating tank system at the University of Texas Health Science Center at Houston Laboratory Animal Medicine and Care on a 14:10-hour light:dark cycle at pH 7.5 and 28°C. Eggs were obtained from natural spawning of adult fish. Fertilized embryos were bleached for 30 s in 0.05% sodium hypochlorite solution (stock 4.00%–4.99%, Sigma-Aldrich) and kept at 30°C on a 14:10-hour light:dark cycle at pH 7.4. Embryos were raised in petri dishes containing E3 buffer (10 mM HEPES, 5 mM NaCl, 0.17 mM KCl, 0.4 mM CaCl_2_, 0.67 mM MgSO_4_, pH 7.4). The 1× E3 medium was prepared with 10 mM HEPES to neutralize the acidic (pH 3) solution that arose after dissolving DSS in standard E3 buffer. Larvae maintained past 6 days post fertilization (dpf) were fed GEMMA Micro 75 (Skretting) until euthanized. The larvae were maintained in 150-mm-diameter petri dishes containing 90 mL of E3 medium.

### Bacterial strains and growth conditions

The bacterial strains and plasmids used in this study are listed in Table 1. All strains were grown at 37°C in Luria-Bertani (LB) broth or on LB agar plates, with ampicillin (200 µg/mL), kanamycin (50 µg/mL), chloramphenicol (35 µg/mL), tetracycline (10 µg/mL), or gentamicin (15 µg/mL), when required.

**TABLE 1:** Bacterial strains and plasmids

Strain or plasmid	Relevant characteristic(s)	Source or reference
Strains		
MG1655	Non-pathogenic lab *E. coli*	
AIEC LF82	Adherent-invasive *E. coli*, parent strain	Torres lab, UTMB
LF82*∆fimH*	LF82 derivative, *fimH* deletion	This study
LF82*∆ibeA*	LF82 derivative, *ibeA* deletion	This study
LF82*∆fimH:fimH*	LF82 derivative, *fimH* complementation	This study
LF82*∆ibeA:ibeA*	LF82 derivative, *ibeA* complementation	This study
*E. coli* DH5α	Used for cloning experiments	
*E. coli* DH5α λpir	Used for complementation	(77)
Plasmids		
pDOC-C	Cloning vector	(78)
pDOC-K	Carries the kanamycin cassette	
pACBSCE	Recombineering plasmid, encodes the I-SceI and the λ-Red proteins	
pME6032:mcherry	Encodes mCherry protein	
pSTNSK-Cm	Tn7 transposase expression vector	(77)
pGpTn7	Cloning vector	

The LF82 deletion strains were generated using recombineering, as previously described (78). Briefly, constructs were generated by amplifying a kanamycin cassette from the plasmid pDOC-K using oligonucleotide pairs that contain at least 45 bp of homology to the DNA immediately upstream and downstream of the target genes (Table 2). The amplified fragment was inserted into the plasmid pDOC-C, and the construct was verified by sequencing (Azenta Life Sciences). The constructed pDOC-C deletion plasmid and the recombineering plasmid pACBSCE were co-transformed into LF82 via electroporation and transformants plated on LB agar containing chloramphenicol, ampicillin, and kanamycin. Selected colonies were grown in LB broth containing 0.5% glucose for 2 hours and then induced with 0.5% arabinose for 4 hours. The cells were then collected by centrifugation and plated on LB agar without NaCl, but containing 5% sucrose and kanamycin. Sucrose-insensitive and kanamycin-resistant recombinant colonies were transferred to LB chloramphenicol plates to confirm loss of the pACBSCE plasmid. Loss of the pDOC-C plasmid was confirmed with pDOC-specific oligonucleotides. Gene deletion was assessed by PCR using primers listed in Table 3.

**TABLE 2:** List of primers used to amplify the pDOC-K plasmid with 45 bp homology to the DNA upstream and downstream of *ibeA* and *fimH^a^*

Gene	Forward primer sequence (5–3)	Reverse primer sequence (5–3)
*ibeA*	CGGAATTCGCGCGGGGGATTGTTTTACTCAATTATTGAAT ACGGAGATAAAGTATGGAA*GACCGGTCAATTGGCTGGAG*	CGGCTAGCGCGCGACATAAAAACTGGGTTTTTCTCTCATAACTTTA TTCCCTGTTAAAA*AATATCCTCCTTAGTTCCTATTCCGAAGTTC*
*fimH*	CGGAATTCTTAGCATCACCTATACCTACAGCTGAACCCGA AGAGATGATTGTAATGAAA*GACCGGTCAATTGGCTGGAG*	CGGCTAGCTCAGGTAATATTGCGTACCTGCATTAGCAATGCCCTG TGATTTCTTTATTG*AATATCCTCCTTAGTTCC*

^*a*^ The restriction site is underlined, and the region homologous to the kanamycin cassette is in italics.

**TABLE 3:** List of primers used to verify deletion mutants

Strain	Forward primer sequence (5–3)	Reverse primer sequence (5–3)
LF82*ΔfimH*	CAACCAAAACAGTTCAGG TGG	GCTGATTATTAGCATGGTAGCG
LF82*ΔibeA*	GGCAAAGAGAGATGATCT CCTT	CCCATAACACCGATGCCAATA

The complementation strains were constructed by insertion of the gene of interest and its endogenous promoter into the respective deletion strains using a Tn7-based vector system (77). Briefly, the genes were cloned in pGp-Tn7-Gm and then introduced into DH5α-λpir by electroporation to construct pGp-Tn7-fimH and pGp-Tn7-ibeA vectors. Positive clones were screened by colony PCR and confirmed by Sanger sequencing. The pGp-Tn7-fimH and pGp-Tn7-ibeA vectors were electroporated into LF82Δ*fimH* and LF82Δ*ibeA* harboring the Tn7-transposase encoding, temperature-sensitive plasmid pSTNSK-Cm. Transformants were plated on LB agar containing gentamicin and chloramphenicol, and then incubated at 30°C for 20 hours. Selected colonies were further streaked on LB agar plates without antibiotics and incubated at 42°C for 20 hours to promote the loss of plasmid pSTNSK-Cm. The colonies were passaged four to five times on LB agar plates (no antibiotic), incubated at 37°C, and screened for resistance to gentamicin and sensitivity to chloramphenicol.

The deletion of *ibeA* or *fimH* and their integration at the attTn7 site was confirmed by PCR (Table 4) and whole-genome sequencing. Genomic DNA was isolated using DNeasy Blood and Tissue kit (QIAGEN, catalog no. 69504) and analyzed by Nanopore sequencing (Plasmidsaurus). Plasmidsaurus also generated a complete genome assembly and annotation. Inspection of those genome assemblies showed that the intended mutations were present in the appropriate strains and that the complementation constructs were correctly integrated at the expected loci. To rule out the possibility that fortuitous mutations were introduced during strain construction, two bioinformatic approaches were used. First, we used Snippy (https://github.com/tseemann/snippy) to compare the nanopore reads to the reference genome (composed of the chromosome https://www.ncbi.nlm.nih.gov/datasets/genome/GCF_021398935.1/ and plasmid https://www.ncbi.nlm.nih.gov/nuccore/NC_011917.1/). Second, we mapped the nanopore reads to the same reference genome using Minimap2 and then used FreeBayes to identify possible single-nucleotide polymorphisms (SNPs). The candidate SNPs identified by either approach were analyzed by inspecting the alignments with IGV (https://software.broadinstitute.org/software/igv/download). This showed that there were no fortuitous mutations that were introduced during strain construction. The *E. coli* strains were electroporated with the mCherry-expressing pME6032 plasmid to visualize the bacteria inside of the zebrafish intestine.

**TABLE 4:** List of primers used to analyze the integration of the Tn7 transposon system at the attTn7 site located downstream of the *glmS* gene

Strain	Forward primer sequence (5-3)	Reverse primer sequence (5-3)
LF82 complementation strain	TGG CTT ACC ACG TTG CGC TG	CAT ACA CCG GCG CAG GGA AG

### Burden of *E. coli* inside of paramecia and larval zebrafish infections

Paramecia were propagated as described (18) 1 day prior to the infection experiment and every 2 weeks to maintain live cultures. Loading of paramecia with AIEC LF82 and MG1655 was conducted as described previously (18). On the day of the experiment, paramecia were co-cultured with either AIEC LF82 or MG1655, and the amount of *E. coli* inside of the paramecia was assessed by lysing the paramecia with 1% Triton X-100 followed by dilution plating and colony forming unit counting, as previously described (18).

*E. coli*-loaded paramecia were counted using an automated cell counter (Life Technologies Countess II), and a final concentration of 2 × 10^5^ paramecia/mL in E3 medium was used to feed the larvae for 2 hours at 30°C in a 6-well sterile plate.

### Bacterial colonization and clearance in larvae

The *E. coli* burden in zebrafish larvae was assessed starting 2 hours post infection (hpi). Briefly, the larvae were anesthetized in the E3 medium with 0.16 mg/mL tricaine and washed six times to remove excess paramecia. Infected zebrafish larvae were euthanized with 1.6 mg/mL of tricaine. The euthanized larvae were then incubated with 100 µL of a 1-mg/mL filter-sterilized pronase solution, vortexed, and placed at 37°C for 6 minutes. The larvae were then homogenized by repeated passage through a 31-gauge needle attached to a 1-mL syringe. In all cases, the samples were serially diluted, and 5 µL of each dilution was plated on CHROMagar O157 plates (DRG International Inc). The plates were incubated at 30°C for 24 hours and then at room temperature for an additional 24 hours to permit full growth of colonies. The number of dark steel-blue (AIEC) and mauve (MG1655) colonies were assessed afterward. Data were analyzed with the GraphPad Prism software, version 9.

### DSS administration and survival analysis of DSS-treated larvae

Colitis grade dextran sulfate sodium (36,000–50,000 MW, MP Biomedical) was used to induce enterocolitis as previously described by others (17). At 3 dpf, 120 larvae were anesthetized with 0.16 mg/mL of tricaine and transferred to a 150-mm-diameter petri dish containing 90 mL of freshly prepared 0.5% (wt/vol) DSS dissolved in E3 medium. The DSS treatment was repeated for three consecutive days. Survival was assessed daily by observing the presence or absence of a heartbeat on anesthetized larvae using an Olympus SZX10 stereomicroscope. Dead larvae were removed, and the survivors were transferred to a new petri dish containing DSS in E3 medium every day following assessment.

### Measurement of intestinal and body length, and swim bladder assessment

All larvae were imaged on an Olympus SZX10 stereomicroscope at 1.6× magnification. Fish were anesthetized in 0.16 mg/mL tricaine and embedded in 1% low-melting agarose (LMA). ImageJ was used for image analysis to assess whole body and intestinal length. The length of the intestine was measured from the beginning of the bulb to the end of the cloaca, and the total body length was determined from the mouth to the tip of the tail. The presence of a swim bladder was visualized under the stereomicroscope on anesthetized larvae. The data were analyzed using GraphPad Prism.

### Histological analysis

Zebrafish larvae were fixed in 4% formaldehyde diluted in PBS and incubated overnight (O/N) at 4°C. Larvae were processed for histological analyses by the UT-Health Core Histopathology Lab. Briefly, larvae were embedded in paraffin, sectioned along the sagittal plane at 2 µm, and stained with H&E. Imaging was performed on an AmScope microscope with an MU1003 camera and the AmScope software version x64, 3.7.11443.20180326.

### Neutrophil and macrophage recruitment

Zebrafish larvae were anesthetized, embedded in 1% LMA in a 6-well glass bottom plate, and imaged on an Olympus Fluoview FV3000 confocal microscope for 3–21 hpi. A *Z*-stack of 190 images of 2 µm slices was analyzed with Fluoview FV31S-SW. The images were then imported into the Imaris software, version 9.7.2, which was used to quantify the number of intestinal GFP-expressing neutrophils or macrophages over the course of 3–21 hpi.

### Immunofluorescence

Larvae were euthanized and placed in a 4% formaldehyde solution O/N at 4°C. Then the larvae were washed twice with 1× PBS, permeabilized in acetone for 15 minutes at −20°C, and incubated in PBDT blocking solution (PBS, 1% BSA, 1% DMSO, and 0.5% Triton-X100) O/N. The larvae were then incubated with anti-α-laminin at a 1:25 dilution (Sigma-Aldrich, L9393) O/N at 4°C. The following day, the samples were washed and incubated with goat anti-rabbit IgG Alexa Flour 488 using a 1:250 dilution (Thermo Fisher Scientific, A27034) and 1 µM/mL DAPI O/N at 4°C. The samples were then washed for 30 minutes, three times with a washing solution (1× PBS, 0.1% Tween-20, and 0.1% Triton X-100). Some larvae were stained with phalloidin (300 units/mL) and 1 µM/mL DAPI. Samples were imaged on a confocal microscope (Olympus Fluoview FV3000 confocal microscope at 60× magnification), and images were transferred to cellSENS version 2.3 for deconvolution with five iterations.

### Quantification of bacteria inside of epithelium

Bacteria inside of the intestinal epithelium were quantified on deconvoluted images taken after immunofluorescence imaging. ImageJ was used to quantify the fluorescent signal of the mCherry channel (representing bacteria) (79). The data were plotted using Graphpad Prism, and significance was determined using a Mann-Whitney *U* test.

### RNA isolation, reverse transcription, and quantitative PCR

RNA was isolated from 15 zebrafish larvae for each condition. Briefly untreated or DSS-treated larvae, fed or unfed paramecia, were euthanized, homogenized in TRIzol reagent (Thermo Fisher, 15596026) using a disposable pellet pestle (Fisher Scientific, 12-141-364), and RNA was extracted using a standard protocol (80). Isolated RNA was treated with RNase-free DNase (Qiagen) and cleaned and concentrated using a Zymo Research RNA Clean & Concentrator Kit. Removal of DNA contamination was verified by PCR using purified RNA as template.

Reverse transcription was carried out using oligo (dT) primers and the SuperScript IV First-Strand cDNA Synthesis Reaction system. The concentration of the cDNA was measured using a Nanodrop-spectrophotometer, and 45 ng of cDNA was used for each reaction. cDNAs and primers (listed in Table 5) were mixed with Luna Universal qPCR Master mix (New England Biolabs), and amplification was carried out in duplicate in a CFX96 Real-Time System C1000 Touch Thermal Cycler (Bio-Rad, Hercules, CA, USA). The *elfα* and *rpl13* genes were used as internal controls, and the relative fold-change for each gene of interest was expressed in 2^−∆∆CT^, where ∆∆CT = [(CT gene of interest − CT internal control) one condition − (CT gene of interest − CT internal control)] another condition (81). For DSS experiments, the DSS data were normalized to the untreated group, whereas in the infection experiments, the data were normalized to control-fed paramecia without added bacteria.

**TABLE 5:** List of primers used to analyze the transcription of pro-inflammatory genes and housekeeping genes

Gene	Forward primer sequence (5–3)	Reverse primer sequence (5–3)
*rpl13*	TCTGGAGGACTGTAAGAGGTATGC	AGACGCACAATCTTGAGAGCAG
*il1b*	ATCAAACCCCAATCCACAGAGT	GGCACTGAAGACACCACGTT
*cxcl8-l1*	TGTTTTCCTGGCATTTCTGACC	TTTACAGTGTGGGCTTGGAGGG
*mmp9*	CATTAAAGATGCCCTGATGTATCCC	AGTGGTGGTCCGTGGTTGAG
*tnfa*	GTTTATCAGACAACCGTGGCCA	GATGTTCTCTGTTGGGTTTCTGAC

## SUPPLEMENTAL MATERIAL

Supplemental Figures msphere.00512-23-s0001.docx Figures S1-S8.

ASM does not own the copyrights to Supplemental Material that may be linked to, or accessed through, an article. The authors have granted ASM a non-exclusive, world-wide license to publish the Supplemental Material files. Please contact the corresponding author directly for reuse.
