# Supplementary material for: Colonization of larval zebrafish (Danio rerio) with adherent-invasive Escherichia coli prevents recovery of the intestinal mucosa from drug-induced enterocolitis
Source: mSphere. 2023 Nov 16;8(6):e00512-23. doi: 10.1128/msphere.00512-23 (PMC10732064; doi:10.1128/msphere.00512-23)
Supplement: Supplemental Figures — Figures S1-S8. [file msphere.00512-23-s0001.docx]

Supporting Information for

**Colonization of larval zebrafish (*Danio rer*io) with adherent-invasive *Escherichia coli* prevents recovery of the intestinal mucosa from drug-induced enterocolitis**

Erika Flores^1,2^, Soumita Dutta^2^, Rachel Bosserman^2,3^, Ambro van Hoof^1,2^, and Anne-Marie Krachler^1,2,*^

^1^Microbiology and Infectious Diseases Program, University of Texas MD Anderson Cancer Center UTHealth Graduate School of Biomedical Sciences, Houston, TX, USA

^2^Department of Microbiology and Molecular Genetics, The University of Texas Health Science Center at Houston, Houston, TX, USA

^3^Current address: Washington University, St. Louis, MO, USA

^*^Corresponding author: [Anne.Marie.Krachler@uth.tmc.edu](mailto:Anne.Marie.Krachler@uth.tmc.edu)

**
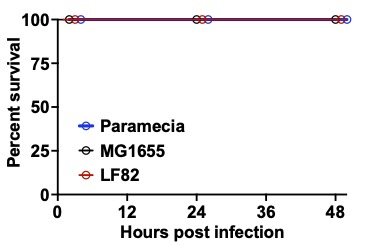
**

**Figure S1. Infection with *E. coli* does not induce mortality in healthy zebrafish larvae.** Survival of untreated larvae that were fed paramecia alone (blue), paramecia containing *E. coli* MG1655 (black) or paramecia containing *E. coli* LF82 (red), n ≥ 15. Kaplan-Meier and Mantel-Cox test. Differences between the three conditions were not statistically significant.

**
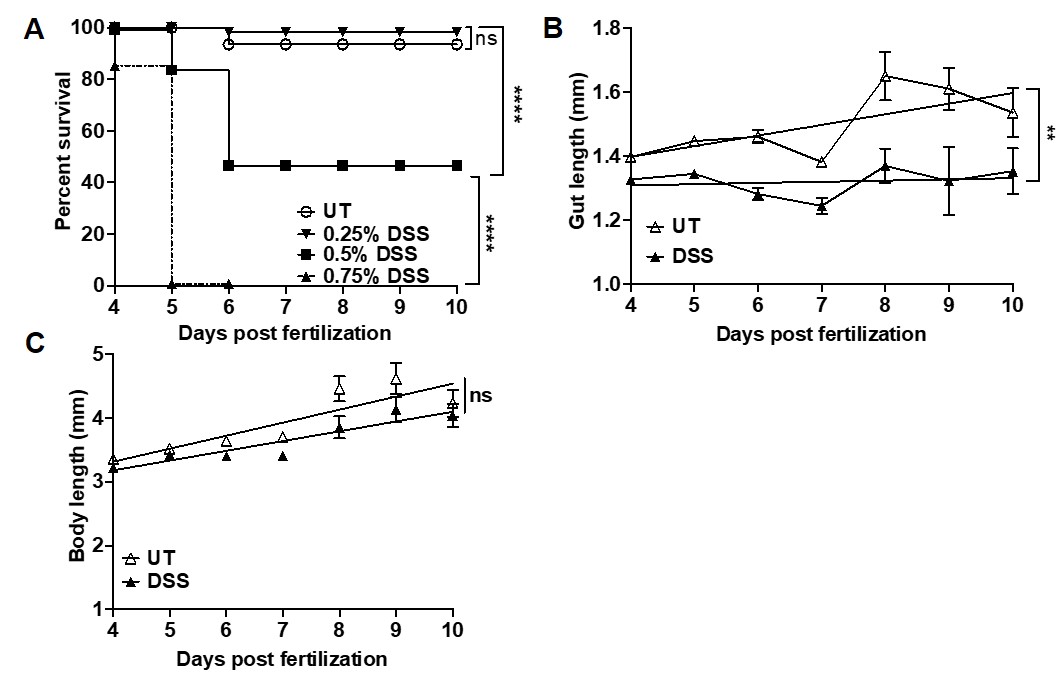
**

**Figure S2. Survival and growth of larvae following DSS treatment. (A)** Survival of larvae administered 0.25%, 0.5%, or 0.75% DSS in buffered E3 medium from 4 to 10 dpf, relative to untreated (UT) controls. Data was analyzed using a Kaplan-Meier plot and Mantel-Cox test. ****, P < 0.0001; n=20 per condition. **(B)** Quantification of the lengths of the intestine and **(C)** whole body of UT and 0.5% DSS treated larvae from 4 to 10 dpf, n≥13. Group differences were analyzed with linear regression. **, P ≤ 0.01; ns, not statistically significant.


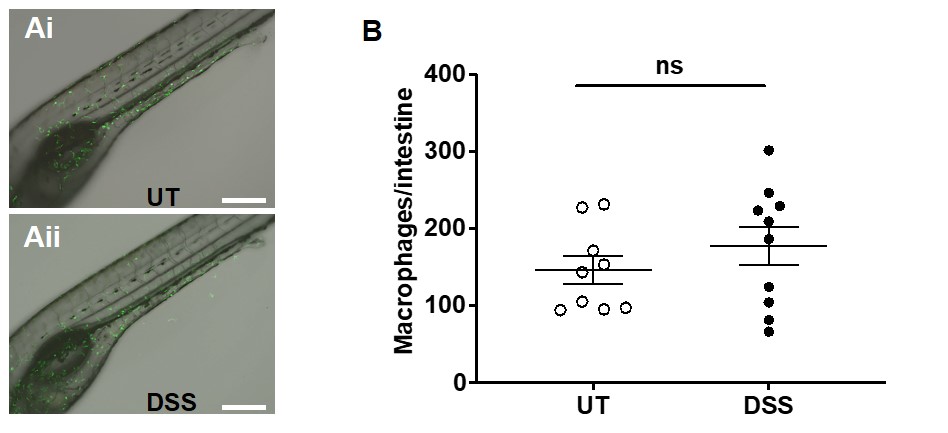


**Figure S3. Macrophage recruitment to the intestine does not change after DSS treatment.**

**(A)** Live imaging of whole-mount untreated **(Ai)** and DSS-treated **(Aii)** larvae with green, fluorescent macrophages; Scale bar represents 200 μm. **(B)** Enumeration of macrophages recruited to the intestine of Tg(*mpeg1*::*egfp*) larvae treated with 0.5% DSS for 3 days and control (UT) larvae. Data presented are individual measurements (n$\leq$10), means and SEM. Unpaired two-tailed t-test; ns, not statistically significant.


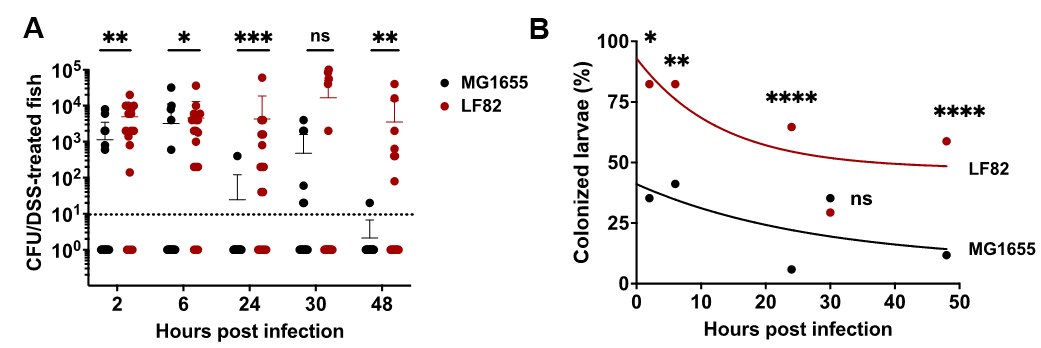


**Figure S4. AIEC LF82 colonizes with a higher burden and is cleared slower than MG1655 in DSS-treated larval zebrafish. (A)** Quantification of *E. coli* MG1655 (black) and LF82 (red) CFUs per fish treated with DSS. Fish that did not have any detectable *E. coli* were annotated as “1 CFU”; n = 17 fish/condition. The detection limit was 10 CFU/fish (black dashed line). **(B)** Bacterial clearance was quantified as % fish with a burden of AIEC and MG1655 above the detection limit from 0-48 hpi. Bacterial clearance was analyzed using a Fisher’s exact test. The half-life of LF82 is 18 hours and 6 hours for MG1655. *, P ≤ 0.05; **, P ≤ 0.01; ***, P ≤ 0.001, ****, P ≤ 0.000.1; ns, not significant;


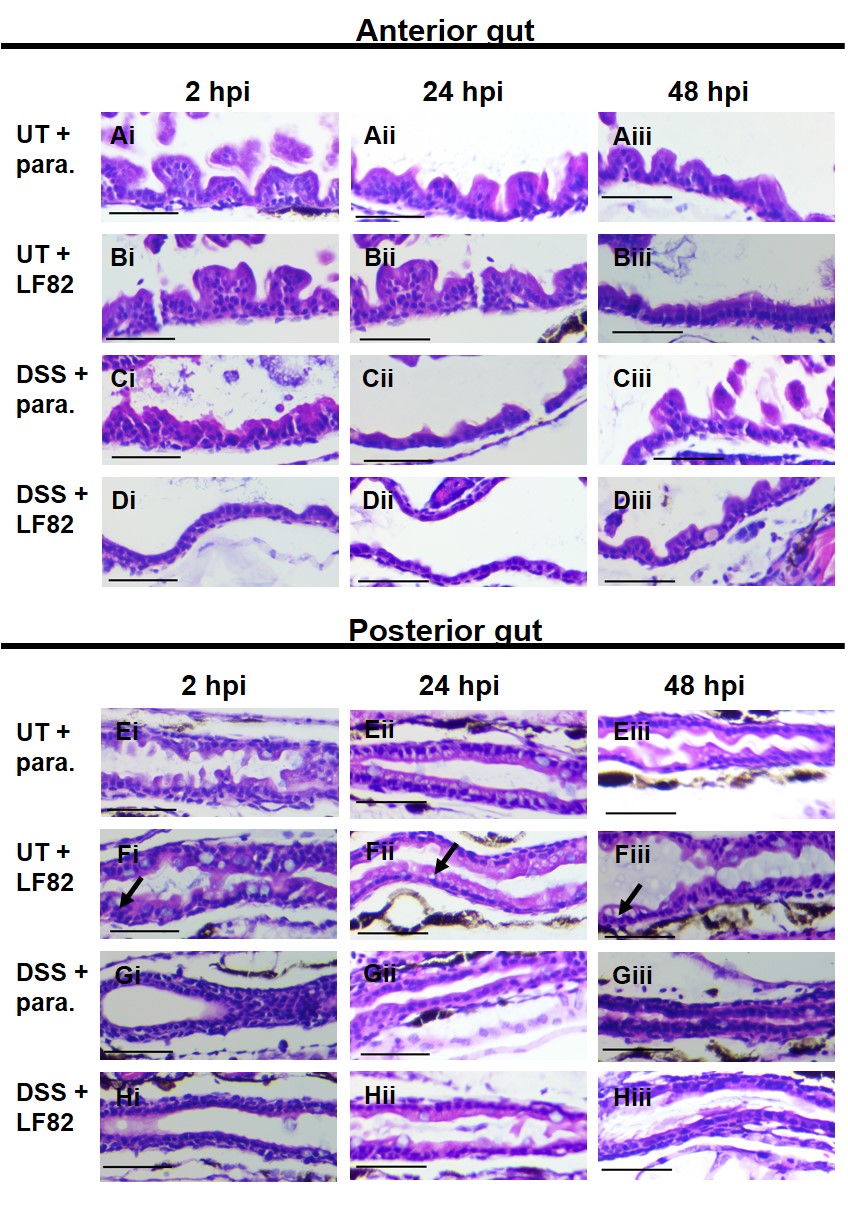


**Figure S5.** Representative (n=4) longitudinal sections of the anterior **(A-D)** and posterior **(E-H)** gut of untreated (UT) larvae fed paramecia (para.) alone, **(A, E)** or UT larvae fed paramecia containing AIEC LF82 **(B, F)**, and DSS-treated larvae fed paramecia alone **(C, G)**, or paramecia containing AIEC LF82 **(D, H)**; Larvae at 2, 24, 48 hpi **(i-iii)**, corresponding to 6, 7, and 8 dpf, respectively. Black arrows point to goblet cells. Scale bars =50 μm;


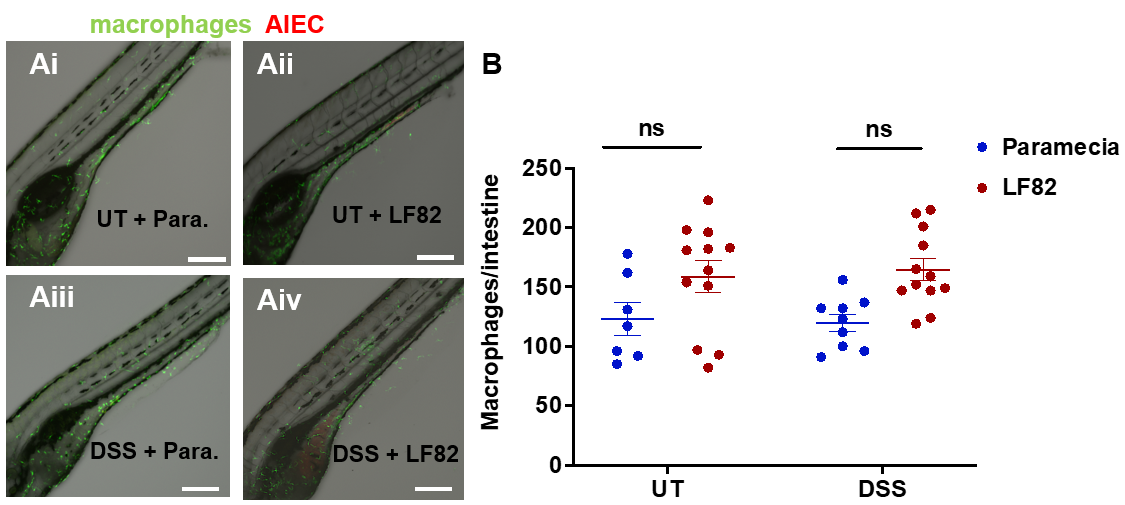


**Figure S6. Macrophage recruitment to the intestine does not change in fish infected with AIEC LF82. (A)** Representative confocal images from live imaging of untreated **(Ai, Aii)** and DSS-treated **(Aiii, Aiv)** Tg(*mpeg1*::*egfp*) larvae fed paramecia vehicle alone **(Ai, Aiii)** or paramecia containing LF82 **(Aii and Aiv).** Macrophage (green), AIEC (red); Scale bars represent 200 μm. **(B)** Enumeration of macrophages recruited to the intestine of untreated or DSS treated larvae fed paramecia alone (blue), or paramecia containing LF82 (red). Data shown are individual measurements from n ≥ 10 fish, means and SEM. Unpaired two-tailed t-test; ns= not significant.


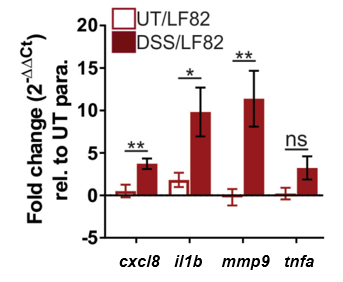


**Figure S7. DSS induced enterocolitis enhances pro-inflammatory response to subsequent LF82 infection.** qRT-PCR analyses of *cxcl8-l1, il1b, mmp9,* and *tnfa* in untreated (white) and DSS-treated (red) larvae infected with AIEC LF82 relative to untreated, paramecia fed controls (1-fold), at 6 dpf; Data are means ± SEM from n=3 independent experiments. Unpaired two-tailed t-test, *, P ≤ 0.05; **, P ≤ 0.01.

**
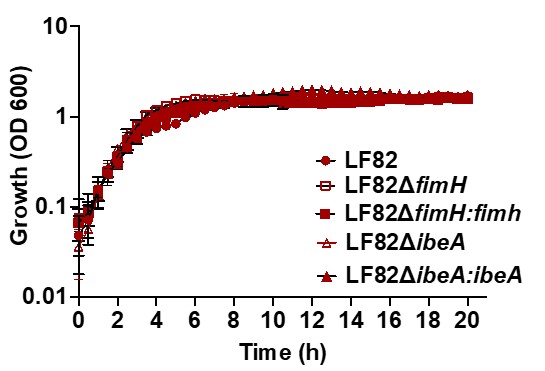
**

**Figure S8. Deletion and complementation of *fimH* and *ibeA* in AIEC LF82 does not affect bacterial growth.** Growth of LF82, LF82Δ*fimH,* LF82Δ*fimH:fimH* complementation strain, LF82Δ*ibeA*, and LF82Δ*ibeA:ibeA* complementation strain, in LB over 20 hours; Data are means ±SEM from n=3 independent experiments.
